# Preservation of Chloroform

**Published:** 1864-08

**Authors:** 


PRESERVATION OF CHLOROFORM.—It requires but a short time for chloroform which is exposed to the sun's rays to undergo decomposition hydrochloric acid being developed, and a strong odour of chlorine being present. This is prevented if the chloroform is kept in the dark; and when it has undergone decomposition by exposure, M. Boettger finds that it may be easily purified by shaking it up with a few fragments of caustic soda. As long, indeed, as it is in contact with the caustic soda it may be preserved for an indefinate period in diffused light.—*Med. Times and Gaz.*, May 28, 1864, from *Bull de Therap.*, May 15.

